# PP74 Are Commonly Used Cost-Effectiveness Thresholds Too Low? Empirical Evidence From Economic Studies On The Value Of Life

**DOI:** 10.1017/S0266462324002435

**Published:** 2025-01-07

**Authors:** Michael Schlander, Diego Hernandez, Oliver Schwarz, Ramon Schaefer

## Abstract

**Introduction:**

In health technology assessment (HTA), “value for money” is frequently conceptualized as incremental cost-effectiveness (CE), with effectiveness measured as (quality-adjusted) life years gained (LYG). Commonly used CE thresholds have been subject to controversial debate. We reviewed and analyzed the worldwide economic literature on the value of a statistical life (VSL), which reported empirical estimates during the last 25 years.

**Methods:**

We conducted an extended systematic literature search in the EconBiz and EconLit databases, spanning the period from January 1995 to December 2020. We transformed VSL data into the implied values of a statistical life year (VSLY) and analyzed the results by regional origin, elicitation method, and annual gross domestic product (GDP) per capita. Then, we performed a regression analysis using the ordinary least squares (OLS) model after log-transforming the VSLY estimates.

**Results:**

We identified 156 empirical economic studies with 169 unique VSL estimates. Overall, the median VSLY was EUR168,367 (mean, EUR256,701) or 6.3 times annual GDP/capita. The median VSLY (expressed as multiples of GDP/capita) differed by regional origin (North America: EUR288,994; 7.2; Europe: EUR168,367, 5.2; Asia: EUR45,260, 4.5) and by elicitation method (revealed preference/wage risk studies: EUR274,625, 9.1; stated preference/contingent valuation studies: EUR113,246, 4.4; stated preference/discrete choice experiments: EUR178,130, 5.2). Regression analyses confirmed that studies with data originating from North America resulted in significantly higher VSLY estimates. The difference remained statistically significant after adjusting for GDP/capita.

**Conclusions:**

The empirical economic literature, while showing heterogeneity by elicitation method, suggests that the adoption of a preference-based or “demand-side” perspective leads to willingness-to-pay estimates for a LYG that exceed commonly used CE thresholds in the context of HTA.

